# Recent Advances and Perspectives on Food-Grade Immobilisation Systems for Enzymes

**DOI:** 10.3390/foods13132127

**Published:** 2024-07-03

**Authors:** Marcella Chalella Mazzocato, Jean-Christophe Jacquier

**Affiliations:** School of Agriculture and Food Science, Institute of Food and Health, University College Dublin (UCD), Belfield, D04 V1W8 Dublin, Ireland; marcella.chalellamazzocato@ucdconnect.ie

**Keywords:** enzyme, enzyme immobilisation, encapsulation, entrapment, covalent attachment, covalent bonding, beverage, enzymatic hydrolysis

## Abstract

The use of enzyme immobilisation is becoming increasingly popular in beverage processing, as this method offers significant advantages, such as enhanced enzyme performance and expanded applications, while allowing for easy process termination via simple filtration. This literature review analysed approximately 120 articles, published on the Web of Science between 2000 and 2023, focused on enzyme immobilisation systems for beverage processing applications. The impact of immobilisation on enzymatic activity, including the effects on the chemical and kinetic properties, recyclability, and feasibility in continuous processes, was evaluated. Applications of these systems to beverage production, such as wine, beer, fruit juices, milk, and plant-based beverages, were examined. The immobilisation process effectively enhanced the pH and thermal stability but caused negative impacts on the kinetic properties by reducing the maximum velocity and Michaelis–Menten constant. However, it allowed for multiple reuses and facilitated continuous flow processes. The encapsulation also allowed for easy process control by simplifying the removal of the enzymes from the beverages via simple filtration, negating the need for expensive heat treatments, which could result in product quality losses.

## 1. Introduction

Enzymes are versatile proteins that act as biological catalysts, accelerating various chemical reactions by converting substrates into products. These molecules have been used in food and beverage production and processing for centuries [1]. In the food industry, they are applied in processes such as bread and cheese manufacture, as well as in beverage production, such as in winemaking and brewing, by either adding exogenous enzymes or promoting endogenous microbial fermentation. In the dairy industry, for example, rennet and β-galactosidase are indispensable enzymes in cheese production for coagulating milk proteins and breaking down lactose, respectively. Similarly, pectinase, xylanase, naringinase, and other enzymes improve fruit and vegetable juice extraction and clarification processes, increasing the production yield and enhancing the final product characteristics by improving the texture and reducing the turbidity and bitterness. Enzymes also offer numerous benefits in beer and wine production, where they can speed up the wort separation and fermentation processes, leading to upgraded filtration results and enhanced flavour, aroma, and stability. In wine production, they are essential in the maceration, juice extraction, and clarification processes [2]. In beer and malted liquor production, enzymes produce sugars during fermentation, control the viscosity, and help in chill-proofing [3]. Additionally, the use of these biocatalytic compounds extends to the production of plant-based dairy-alternative beverages. They are also increasingly used in water treatment and food waste conversion processes to create high-value products such as sweeteners and prebiotic compounds [4]. Therefore, enzymes play an essential role in the food and beverage processing sector, providing high efficiency in converting raw materials into products with desirable characteristics [5] and representing novel alternatives to chemical or mechanical methods for improved yields and quality in the beverage industry.

Nevertheless, their industrial use is often limited due to their low thermal and chemical stability, which restrict their use to a narrow range of pH and temperature values, making them unsuitable for specific applications in the food and beverage industry, such as juice production and winemaking, which are often carried out under acidic conditions or involve pasteurisation processes. Additionally, their high cost, single-use nature, and heat treatment requirements pose further obstacles, making their use complex and expensive. Enzymes are also typically used in batch production systems, which impede efforts to transition to continuous processing, expand the processing scale, and enhance the level of productivity, in addition to the significant challenges in their recovery and reusability that would make the process more cost-effective [1,6,7]. Meanwhile, immobilisation is a practical approach to address these issues for improved enzyme stability and reuse.

The immobilisation techniques involve physically confining enzymes to a specific region while preserving their catalytic capabilities [8]. This method enables the use of enzymes in optimal microenvironments and conditions. It enhances their properties by modifying various catalytic features such as the enzyme specificity, selectivity, and stability across a range of pH and temperature conditions and inhibitor resistance levels, ensuring recyclability over multiple catalytic cycles [1,9]. Additionally, stable immobilised biocatalyst systems simplify the process of enzyme separation from the reaction medium, mitigate or completely prevent product contamination, enable continuous operations, and facilitate the application of enzymes to diverse reactor types [4,10,11]. The potential advantages and suitability of immobilised enzymes in the food industry have prompted numerous investigations in this field. From 2000 to 2023, more than 1800 articles relating to enzyme immobilisation were published and catalogued on the Web of Science, with analytical chemistry (417 articles), food science technology (414 articles), biotechnology and applied microbiology (395 articles), applied chemistry (234 articles), biochemistry and molecular biology (215 articles), and electrochemistry (197 articles) being the most prevalent science and engineering categories mentioned within that database.

**Figure 1:**
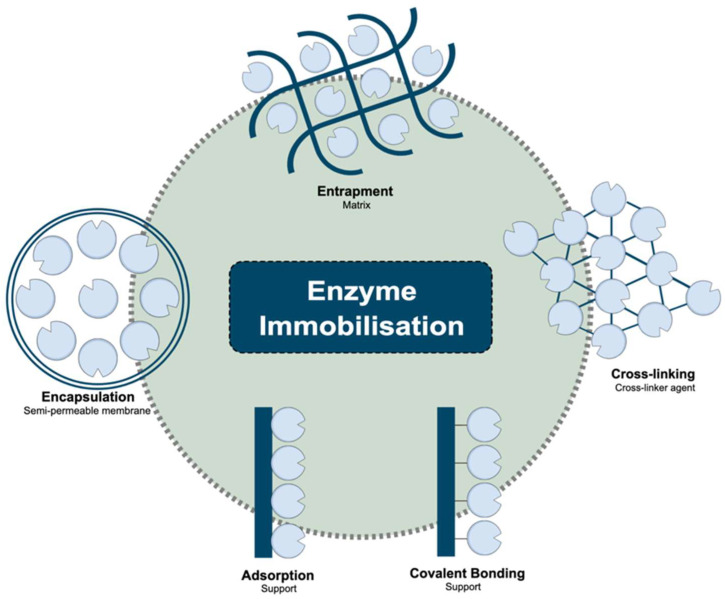
Schematic representation of the main methods of enzyme immobilisation.

The extensive literature can also be divided according to the immobilisation strategy that is employed. As Figure 1 shows, the principal methodologies for the immobilisation of enzymes vary from reversible physical adsorption and ionic linkages to irreversible covalent bonds and physical entrapment into a capsule or polymeric gel. Adsorption, for example, is a reversible immobilisation method. It consists of the attachment of enzymes to solid supports through weak attractive forces (Van der Waals forces, hydrophobic bonding or hydrogen bonding) between the enzyme and the support material to create an enzyme–support complex [12]. The enzymatic solution reacts with the solid support for a while under suitable conditions to allow the attachment, resulting in a high-loading enzyme. Then, the unreacted enzyme molecules are washed with a buffer solution to remove them from the surface. The enzyme–support bond is weak and characterised by poor stability, which may cause a loss of enzyme molecules during their use or washing [13]. Encapsulation and entrapment, in turn, both involve confining enzymes. In entrapped systems, the enzymes are confined into a continuous semipermeable gel or a polymer matrix, while both the substrate and products can diffuse in and out of this structure [1]. Similarly, the encapsulation of enzymes refers to enclosing the catalytic compound in a separate liquid phase within a semipermeable membrane or coat. The semi-permeable barrier also allows substrate and product exchange but stops the enzyme from diffusing. The encapsulation is achieved using techniques such as emulsion, coacervation, and microfluidics [14].

Covalent attachment is another enzyme immobilisation technique—one of the most popular among beverage applications due to its stability and outstanding performance. It involves strong and irreversible covalent bond formation between the enzyme molecules and the support matrix to form a stable complex. The attachment occurs through various chemical reactions according to the functional groups of enzymes and the carrier material, such as the amino group, carboxylic group, phenolic group, sulfhydryl group, and so on. The binding procedure consists of two stages. The first stage involves surface activation using linker molecules such as glutaraldehyde, genipin, or carbodiimide. In the latter stage, enzymes are added for covalent coupling to the activated support [13]. Equally to the adsorption technique, the catalytic compounds are located and attached on the surface of the support but linked by a stronger and permanent bond. Lastly, the cross-linking immobilisation technique is an irreversible method performed using intermolecular cross-linkages between enzyme molecules. This technique requires a cross-linking agent, which links the enzyme molecules together in a three-dimensional aggregate and is a carrier-free enzyme immobilisation system. The cross-linked enzyme aggregates (CLEAs) are easily prepared and involve enzyme precipitation from aqueous solutions by adding organic solvents, salts, or non-ionic polymers [15].

**Figure 2:**
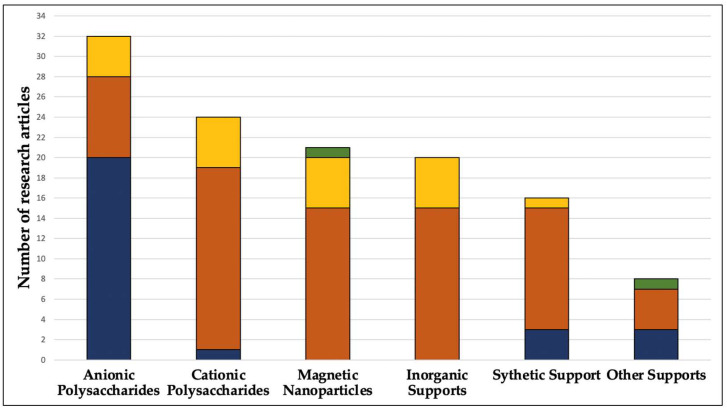
Distribution of research articles dealing with enzyme immobilisation techniques for the production of beverages in studies catalogued on the Web of Science from 2000 to 2023 according to the type of material applied as the matrix or support. Each colour represents a method of immobilisation: (■) covalent attachment; (■) enzyme confinement (entrapment and encapsulation); (■) physical adsorption; (■) enzyme cross-linking.

The nature of the matrices or supports is another essential parameter during immobilisation. An appropriate material is crucial and significantly impacts the properties of the enzyme and its catalytic capacity. The geometry, size, pore diameter, specific surface area, and activation degree of the support are some parameters that define its suitability in the system [4]. Hydrogels, polymers, and inorganic materials are examples of common carrier matrices or supports for enzyme immobilisation. Apart from its affordability, an ideal carrier must possess characteristics such as inertness, stability, physical strength, and the ability to increase the enzyme’s specificity or activity with reduced product inhibition, as well as the ability to prevent microbial contamination [16]. The immobilisation of commercial enzymes, especially for biomedical, food, and pharmaceutical uses, must also involve low-cost, non-toxic, and sometimes biodegradable matrices. Many matrix alternatives are available for enzyme immobilisation, including organic, inorganic, natural, and synthetic alternatives [7]. Chitin, chitosan, and cellulose are widely applied for adsorption and covalent binding purposes, while agar, agarose, alginate, gelatin, cellulose, and polyacrylamide are biopolymers that have been used for entrapment. Likewise, inorganic matrices are good options and offer several advantages However, they are expensive compared to organic matrices and require other chemical substances, which might increase the cost of the procedure [7]. Figure 2 illustrates the distribution of enzyme immobilisation techniques based on the type of material employed as a matrix or support. The data presented in the figure were derived from an extensive analysis of articles that explored enzyme immobilisation methods and used these systems specifically in beverage applications from 2000 to 2023. As observed, covalent attachment has emerged as the most frequently utilised method for immobilising enzymes for beverage applications. Among the various supports, cationic polysaccharides, magnetic nanoparticles, and synthetic materials have proven to be the predominant carriers for this technique. Other extensively investigated methods include entrapment and adsorption. Anionic polysaccharides have notably led the way in enzyme entrapment. In contrast, the adsorption technique has been employed across a broad spectrum of anionic and cationic polysaccharides, magnetic nanoparticles, and other inorganic supports.

The immobilisation of enzymes is a complex and extensive subject that offers significant advantages in the food and beverage industry, offering enhanced enzyme performance and expanding their applications. The ongoing research and development efforts in this field are promising for advancing the use of bioprocessing technology, potentially resulting in increased productivity, cost-effectiveness, and high-quality food and beverage products. This literature review aimed to analyse approximately 120 articles published and catalogued on the Web of Science between 2000 and 2023. The studies focused on developing enzyme immobilisation systems for beverage processing applications. The review evaluated the impacts of immobilisation on the enzymatic activity, including its effects on the chemical and kinetic properties, recyclability, and feasibility of applying immobilisation systems in continuous processes. Furthermore, this paper compiles the principal applications of immobilised enzymes in the beverage sector, identifies their prospects, and discusses the limitations of the immobilisation techniques.

## 2. Methodology for Collecting and Screening the Literature

**Figure 3:**
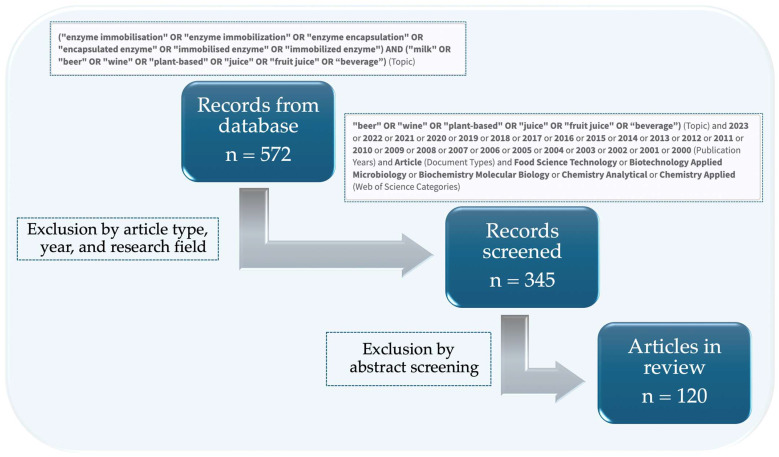
Flow chart of the study selection process.

A systematic literature review aims to identify, evaluate, and interpret relevant research papers on a particular issue, thematic area, or phenomenon of interest [17]. For this review, our systematic approach to data collection is presented in Figure 3. Following the PRISMA (Preferred Reporting Items for Systematic Reviews and Meta-Analysis) protocol for systematic reviews, the literature search was conducted on the Web of Science database to collect relevant articles until March 2024, using the advanced search options. The Web of Science database was selected due to its extensive and comprehensive coverage of high-quality research articles in the fields of food science and technology.

Search terms such as “enzyme immobilisation” and “immobilised enzyme” together with “milk”, “beer”, “wine”, “plant-based”, “fruit juice”, and “beverage” were used to limit the search to papers dealing with the application of immobilised enzymes in beverage processing. In addition, the search results were filtered by year (from 2000 to 2023), document type (scientific article only), and research field (food science technology, applied biotechnology, microbiology, biochemistry, molecular biology, analytical chemistry, and applied chemistry). Then, to emphasise how immobilisation can change the properties of enzymes and benefit beverage production, the articles that did not approach those topics, specifically those related to sensor development using immobilised enzymes, were excluded.

## 3. Immobilisation to Change Enzyme Properties

Each enzyme operates optimally within a specific pH and temperature range, where its catalytic activity peaks. Any deviation from this ideal range, either in pH or temperature, decreases the enzymatic activity. Similarly, the enzyme’s efficiency decreases when the temperature surpasses the optimal level. These characteristics pose significant limitations in the food and beverage industry, as enzymes can undergo denaturation when exposed to high temperatures or unfavourable pH conditions.

To address these limitations, enzyme suppliers strongly advocate introducing modifications to the enzyme structure to enhance the thermal and pH resistance. Techniques involving the genetic modification of the enzyme source, stabilisation of multimeric enzymes through chemical cross-linking, protein engineering, and enzyme immobilisation procedures are employed to improve the enzymatic properties [18]. Among these techniques, enzyme immobilisation has emerged as a superior alternative to the conventional use of enzymes, offering several advantages. It provides more resistant and commercially attractive enzymes and a revolutionary way to terminate reactions by easily removing the biocatalytic compound from the medium.

### 3.1. Optimum pH and pH Stability

The pH resistance of the enzymes can be enhanced via the use of immobilisation in a charged microenvironment. This microenvironment can affect the enzyme’s active site and alter the properties of the immobilised enzyme [19]. The stability and optimal pH for the maximum reaction rate may vary depending on the surface charge and the nature of the support material. The interaction between the immobilisation support and enzyme molecules, whether hydrophilic or hydrophobic, can cause a shift in the optimum pH compared to native enzymes [20]. This phenomenon, known as the ion partitioning effect, is responsible for the arrangement of ions in an aqueous system due to variations in spatial permittivity.

This literature review on immobilised enzymes used in beverage processes revealed changes in pH behaviour between free and immobilised forms of the enzyme. Table 1 presents some of the findings, indicating variations in the optimum pH. The immobilisation process led to increased, decreased, or unchanged pH levels for optimal enzyme performance. Contrary to expectations, no consistent trend was observed in optimum pH levels based on factors such as the type of immobilisation material, enzyme type and source, or immobilisation technique. For instance, different tendencies for the optimum pH were observed when using a calcium alginate material for entrapment in the immobilisation of xylanase and pectinase. A decrease in optimum pH from 5.0 to 3.0 and an increase of 0.3 units of pH (the optimum pH varied from 4.1 to 4.3) were observed for free and immobilised xylanase and pectinase, respectively [19,21]. Similarly, variations in optimum pH were observed even when keeping the enzyme and source constant but changing the immobilisation technique or material. Among the reviewed articles, 30% showed a slight increase in optimum pH, 26% exhibited a decrease, and 44% showed no change. In most cases, the optimum pH for immobilised enzymes remained slightly higher or lower than that of the native enzyme. Nonetheless, five studies reported a shift to the opposite pH condition, from basic to acidic or from acidic to basic, after the immobilisation of enzymes on the surfaces of chitosan beads [22,23,24], magnetic nanoparticles [25], and zirconium-treated pumice [26]. Additionally, the majority of the studied enzymes performed better in acidic conditions (75.6%), which aligns with the acidic nature of many beverages that undergo enzymatic hydrolysis processes, such as fruit juice, wine, and beer. Therefore, enzymes that exhibit satisfactory performance at low pH levels are desirable.

In contrast to the variations in optimum pH, immobilisation generally improved the pH stability and expanded the pH range in which the enzyme remained active, as demonstrated in Table 1. Immobilisation provides a more stable environment by attaching or confining the enzyme, protecting it from denaturation or inactivation under harsh pH conditions. As a result, immobilised enzymes tend to exhibit greater resistance to extreme alkaline or acidic pH conditions and show a broader range of pH stability compared to native enzymes.

### 3.2. Optimum Temperature and Thermal Stability

Several aspects influence the effect of immobilisation on the enzymatic performance and optimum temperature for enzyme activity. These include the immobilisation method, carrier material, and source and function of the enzyme, which can lead to different outcomes in terms of the enzyme structure, stability, and activity after immobilisation [46]. Additionally, the experimental conditions, such as the carrier material composition and concentration, choice of cross-linking agent, and reaction time for immobilisation system development, can introduce variability, affect the enzyme protection properties, and potentially alter the optimum temperature. Therefore, similar to the optimum pH, the optimum temperatures also varied across the studies reviewed in the literature.

Some immobilisation systems have shown increases in optimum temperature, while others have demonstrated no change. However, among the 120 articles reviewed, just a few studies, which employed organic immobilisation materials, such as alginate [19,42,47], chitosan [6,23,48,49], green coconut fibre [50], and spent coffee grounds [51], reported a decrease in the optimal activity temperature.

Other studies showed varied optimum temperatures even with the same immobilisation method, carrier, and enzyme function but from different sources. For instance, α-galactosidase from *Aspergillus oryzae* [47] and *Bacteroides thetaiotaomicron* [52] was entrapped into calcium alginate. While the former study showed a decrease in the maximum activity temperature after immobilisation (from 50 °C to 45 °C), the latter found no change (60 °C). Similarly, divergent results were observed in the studies conducted by Hackenhaar et al. [53] and de Freitas et al. [54] when immobilising β-galactosidase from *Bacillus circulans* and *Kluyveromyces lactis*, respectively, through covalent attachment onto glutaraldehyde-activated chitosan beads. The first group observed no impact on the optimum temperature (40 °C) [53], while the latter study showed a 10 °C increase in the optimum reaction temperature [54].

Further variation in the optimum temperatures was observed when evaluating the entrapment of identical enzymes (α-galactosidase from *Aspergillus oryzae*) into different immobilisation materials. The system developed with calcium alginate beads resulted in a decrease in the optimum temperature from 50 to 45 °C [47], while the use of κ-carrageenan gel beads cross-linked with glutaraldehyde led to an increase in the optimum temperature from 50 °C to 53 °C [55]. Likewise, the immobilisation of the same enzyme onto zinc oxide nanoparticles [56] or a nanosilver-reduced graphene oxide nanocomposite [57] exhibited different trends in terms of the optimum temperature. An increase in optimum temperature from 50 to 60 °C [56] and no change (50 °C) were observed [57].

The optimum temperatures for the immobilised enzymes also varied depending on their function and source. Studies using the adsorption technique on chitosan beads showed diverse behaviours. Protease immobilisation increased the optimum temperature from 60 to 70 °C [32], while pectinase led to no change [58] and β-glucosidase immobilisation decreased the optimum temperature from 75 to 70 °C [49]. Similarly, studies in which enzymes were entrapped into alginate beads showed varied optimal temperatures for enzymes such as α-galactosidase isolated from peanuts [59], tannase (*Penicillium rolfsii*) [21], pectin methyl esterase (*Lycopersicon esculentum*) [60], xylanase (*Aspergillus flavus*) [61], pectinase (*Aspergillus aculeatus*) [19], and glucose oxidase (*Aspergillus niger*) [30].

The immobilisation of enzymes not only affects their optimal temperature but also significantly influences their thermal stability, a crucial factor in expanding the applicability of enzymes by preserving them against denaturation [48]. The enzymatic activity highly depends on the enzyme’s structure and protein’s conformation. The immobilisation of enzymes can induce structural modifications and create a more rigid microenvironment around the enzyme, which restricts movement and reduces conformational changes. This protects the enzyme from unfolding, denaturation, or aggregation at high temperatures [32]. The stabilisation occurs through enhanced interaction between the enzyme and matrix, facilitated by the use of solid supports and immobilisation techniques that induce molecular rigidity [62]. Techniques such as covalent attachment and cross-linking further increase the enzyme’s conformational rigidity, requiring higher activation energy levels for thermal denaturation reactions and preventing conformational inactivation [33]. The use of glutaraldehyde, for example, in enzyme immobilisation leads to improved thermal stability by introducing additional linkages that restrict enzyme movement at higher temperatures [62]. This enhanced thermal stabilisation has been extensively reported, showing positive outcomes, such as improved stability at elevated temperatures, enzymatic activity retention, and improved performance across a wide temperature range or extended reaction periods [23,32,43,63,64,65]. Such benefits hold significant economic value, particularly in the beverage industry for products derived from enzyme immobilisation, by expanding the utilisation of enzymes.

### 3.3. Kinetic Properties

The use of immobilisation can alter an enzyme’s behaviour (Table 2) by decreasing its conformational changes and increasing its structural rigidity, which affects its kinetic properties [32,66]. Studies indicate that enzyme immobilisation generally reduces the maximum reaction rate (V_max_) while increasing the Michaelis–Menten constant (K_M_). These changes can be attributed to the interaction between the enzyme and functional groups on the support or matrix surface. The immobilisation material may limit access to the enzyme’s active site or restrict the protein flexibility required for substrate binding, thereby reducing the enzyme’s affinity for the substrate [66,67]. Additionally, the formation of a rigid and compact structure creates a physical barrier, hindering mass transfer and reducing the substrate’s availability. Consequently, there are decreases in catalytic activity and efficiency compared to the free enzymes [44,68].

Several factors, including the immobilisation material, porous matrices, and type of binding, significantly impact the changes in an enzyme’s kinetic parameters. The binding methods affect the enzyme’s secondary and tertiary structures, potentially altering its affinity for the substrate [69,70]. Covalent bonds, for instance, although more stable, tend to have a substantial influence on the enzyme’s structure. While adsorption interactions induce minor changes in the Michaelis–Menten constant (as seen in Table 2). A study comparing glutaraldehyde-activated magnetic particles and CLEA showed that the immobilisation methodology affects the enzyme’s kinetics [44]. The use of CLEA resulted in higher K_M_ values compared to glutaraldehyde-magnetic particles. These differences may be attributed to the arrangement of enzymes within the CLEA structure, where active sites may be embedded inside the structure, whereas in the glutaraldehyde-magnetic particles, the enzymes tend to be immobilised on the support surface due to the small pore size, facilitating substrate access and consequently reducing the K_M_.

The influence of cross-linking agents on the kinetic properties was also observed. Treatments involving glutaraldehyde showed less impact on the K_M_ and V_max_ values due to the introduction of a longer chain, which reduced the structural rigidity and flexibility [71]. In a study by Hosseini et al. [72], the kinetic parameters of immobilised pectinase on glass beads cross-linked with poly-aldehyde pullulan or glutaraldehyde were compared. Both cross-linking agents resulted in higher K_M_ and lower V_max_ values compared to the soluble enzyme. However, pectinase cross-linked with poly-aldehyde pullulan exhibited better resistance to substrate diffusion, leading to hindered substrate access to the enzyme’s active site (higher K_M_) compared to the glutaraldehyde cross-linking. This difference was attributed to the more complex structure of poly-aldehyde pullulan, which decreased the enzymatic activity.

**Table 2:** A comparison of enzymatic kinetic properties between the immobilised and free forms of enzymes. Information such as enzyme type and source, the immobilisation method, carrier material, substrate, and beverage application are also displayed. Data in bold represent systems that presented improvement in kinetic properties after immobilisation.

Enzyme and Source	Method	Carrier/Support	Substrate	K_M_		V_MAX_		Application	Ref.
				FE	IE	FE	IE		
Alkaline protease (*Bacillus licheniformis*)	Covalent attachment	Eupergit CM	Casein	26.53 g/L	37.59 g/L	**2.84 g/L.min**	**3.31 g/L.min**	Lactose hydrolysis.	[67]
β-Galactosidase (*Kluyveromyces lactis*)	Adsorption	Polyvinyl-alcohol-functionalised gold nanoparticles	ONPG *	3.56 mmol/L	3.74 mmol/L	2.8 mmol/L.min	2.07 mmol/L.min	Lactose hydrolysis.	[68]
β-Galactosidase (*Kluyveromyces lactis*)	Covalent attachment	Collagen–glutaraldehyde	ONPG *	3.86 mmol/L	7.50 mmol/L	42.92 mmol/L.min	32.37 mmol/L.min	Lactose hydrolysis.	[71]
Pectinase and cellulase (commercial)	Covalent attachment	Glutaraldehyde-activated magnetic particles	Pectin	17.60 mmol/L	25.65 mmol/L	40.58 μmol/min	26.66 μmol/min	Grape juice clarification.	[44]
Pectinase and cellulase (commercial)	Cross-linking	Magnetic CLEA	Pectin	17.60 mmol/L	33.83 mmol/L	40.58 μmol/min	26.87 μmol/min	Grape juice clarification.	[44]
Polygalacturonase (*Aspergillus niger*)	Adsorption	Calcium alginate microspheres	Pectin	4.472 mg/mL	5.041 mg/mL	0.214 U	0.112 U	Apple juice clarification.	[73]
β-Glucosidase (*Bacillus subtilis*)	Covalent attachment	Functionalised silicon oxide nanoparticles	pNPG *	0.9 mmol/L	1.074 mmol/L	3.5 U/mg	1.513 U/mg	Sugarcane juice clarification.	[74]
Pectinase (commercial)	Covalent attachment	Polyaldehyde-pullulan-activated glass beads	Pectin	-	11.2 mg/mL	-	2.2 μmol/min	Barberry juice clarification.	[38]
Pectinase (commercial)	Covalent attachment	Glutaraldehyde-activated glass beads	Pectin	-	10.1 mg/mL	-	2.9 μmol/min	Barberry juice clarification.	[38]
Naringinase (*Penicillium decumbens*)	Covalent attachment	Glutaraldehyde-activated chitosan beads	Naringin	2.56 mmol/L	6.59 mmol/L	1.21 μmol/L.min	0.19 μmol/L.min	Debittering grape juice.	[48]
Protease (*Penaeus vannamei*)	Adsorption	Chitosan nanoparticles	Casein	2.5 μmol/L	2.7 μmol/L	87 μmol/L.min	83 μmol/L.min	Pomegranate juice clarification.	[32]
Pectinase	Covalent attachment	Trichlorotriazine-functionalised polyethylene-glycol-grafted magnetic nanoparticles	Polygalacturonic acid	14.89 mg/mL	10.5 mg/mL	**0.578 U/mL**	**1.190 U/mL**	Pineapple juice clarification.	[75]
β-Galactosidase (*Aspergillus oryzae*)	Adsorption	Nanosilver-reduced graphene oxide nanocomposite	ONPG *	**0.5 mmol/L**	**0.44 mmol/L**	**0.031 mmol/L.min**	**0.039 mmol/L.min**	Lactose hydrolysis.	[57]
Pectinase (*Aspergillus aculeatus*)	Covalent attachment	Montmorillonite support activated with glutaraldehyde	Pectin	**11.49 mg/mL**	**6.06 mg/mL**	2.93 mmol/L.min	1.73 mmol/L.min	Pineapple juice clarification.	[76]
Invertase (bakery yeast)	Entrapment	Poly(VP-co-BAc-co-NHMAAm) film	Sucrose	**29.6 mmol/L**	**4.5 mmol/L**	13.43 μmol/min	13.04 μmol/min	Sucrose determination in fruit juice.	[77]
Papain (*Carica papaya*)	Covalent attachment	Glutaraldehyde-poly(HEMA)–chitosan cryogels	Casein	**4.255 mg/mL**	**1.544 mg/mL**	0.554 mmol/min	0.199 mmol/min	Apple juice clarification.	[43]
Xylanase (*Thermomyces lanuginosus*)	Covalent attachment	Trichlorotriazine-functionalised polyethylene-glycol-grafted magnetic nanoparticles	Xylan	25.51 mg/mL	40.42 mg/mL	**2.69 U/mL**	**6.01 U/mL**	Pineapple juice clarification.	[25]
Laccase (*Pleurotus ostreatus*)	Adsorption	Poly(methacrylate) beads	ABTS	**0.063 mmol/L**	**0.032 mmol/L**	-	-	Fruit juice clarification.	[78]
β-glucosidase (commercial)	Entrapment	Calcium–alginate beads	Cellobiose	**0.22 mmol/L**	**0.21 mmol/L**	**2.52 μmol/mg.min**	**3.35 μmol/mg.min**	Wine aroma enhancement.	[79]
Pectinase (*Aspergillus aculeatus*)	Covalent attachment	Polyethyleneimine-based cryogel	Starch	**40 mg/mL**	**31.25 mg/mL**	66.6 mg/mLmin	1 mg/mLmin	Apple juice clarification.	[80]
β-Glucosidase (*Laminaria hyperborea*)	Covalent attachment	Chitosan glutaraldehyde beads	Cellobiose	0.18 mmol/L	0.21 mmol/L	3.6 μmol/min.mg	3.5 μmol/mg.min	Aroma hydrolysis in grape must.	[49]
β-galactosidase (*Escherichia coli*)	Covalent attachment	Magnetic graphene oxide nanocomposites	ONPG *	6.99 mmol/L	8.50 mmol/L	47.8 mmol/L.min	38.2 mmol/L.min	Lactose hydrolysis.	[34]
Invertase (Baking yeast)	Covalent attachment	Poly(N-vinylpyrrolidone-*co*-butylacrylate-*co*-N hydroxymethylacrylamide) terpolymer membranes	Sucrose	**29.41 mM**	**8.33 mM**	13.4 μM/min	12.2 μM/min	Hydrolysis of sucrose in peach juice and orange juice.	[81]

* ONPG: o-nitrophenyl β-d-galactopyranoside; pNPG: 4-nitrophenyl β-D-glucopyranoside.

The attachment of enzymes on surfaces can enhance the active site availability by improving their accessibility to substrates and lowering the K_M_ values [43,57,76,77,78,79]. Some studies have observed a positive effect on V_max_ values, indicating an improvement in the catalytic activity [25,57,69,75]. However, the impact on the enzyme activity can be influenced by various factors, including the immobilisation method, substrate nature, and enzyme characteristics. Therefore, it is crucial to consider the unique characteristics of each enzyme–substrate system and the specific conditions of the immobilisation process, such as the surface chemistry of the enzyme and support and their interaction, to predict and prevent potential adverse effects on the catalytic and kinetic activities of the enzyme. In addition, it is essential to note that improving the K_M_ values does not necessarily result in superior V_max_ values.

In addition, there are several strategies to overcome the potential decreases in the efficiency of the kinetic parameters. Increasing the substrate concentration can help achieve the maximum reaction rate. Moreover, using substrates with simpler or smaller molecules can facilitate access to the enzyme. Selecting cross-linking agents that produce longer chains between the enzyme and support is another way to minimise the loss of substrate affinity [33,44,71,72,73]. Enzymes immobilised on the surface allow simplified substrate access and circumvent the negative impacts on the kinetic properties, as well as the utilisation of a low concentration of immobilisation material [69].

### 3.4. Thermodynamic Properties

Thermodynamic properties such as the activation enthalpy (ΔH°), Gibbs free energy (ΔG°), and entropy of activation (ΔS°) are fundamental in assessing an enzyme’s stability and functionality across varying conditions. These parameters, derived from kinetic constants (kd and Ed), provide crucial insights into the energy requirements and spontaneity of enzyme inactivation processes, and can provide relevant information regarding the enzyme immobilisation systems [82].

Ahmed et al. [83] investigated these thermodynamic properties in the hydrolysis of casein by protease immobilised on glass–ceramic inorganic supports. Their findings revealed that the immobilised enzyme exhibited a higher enthalpy value than the free enzyme (by 1.1-fold at 70 °C), indicating enhanced resistance to high temperatures attributed to conformational changes induced by immobilisation of the adsorbed enzyme. Similarly, the glass–ceramic-immobilised protease displayed slightly higher entropy values (−0.27 J/mol/K) compared to its free form (−0.26 J/mol/K), indicating reduced spontaneity at the tested temperatures. Moreover, the Gibbs free energy value for the immobilised enzyme was positive and 1.0-fold higher than that for the free enzymes, indicating their greater thermal stability [83].

In another study, cellulase immobilised on iron oxide nanoparticles exhibited significant enhancements in thermal stability compared to the free enzyme [82]. The activation energy increased from 91.26 ± 2.28 kJ/mol for the free enzyme to 107.19 ± 3.03 kJ/mol for the immobilised form at temperatures ranging from 55 to 75 °C, indicating a greater energy requirement for denaturation and suggesting enhanced stability. The enthalpy values for the immobilised cellulase were consistently higher and decreased with increasing temperature, resulting in lower energy requirements for the denaturation of the free enzyme compared to the immobilised form. Both results suggest increased stability of the immobilised enzyme under thermal stress. The Gibbs free energy of the immobilised cellulase was marginally higher than for the free enzyme, suggesting additional resistance to thermal unfolding. The negative entropy values for both the free and immobilised cellulase forms indicated reduced disorder in the enzyme’s structure post-immobilisation [82].

Further investigations have demonstrated promising outcomes with immobilised enzymes such as laccase over multi-walled carbon nanotubes [84], as well as an amylase covalent attached on sodium alginate carriers [85]. The authors found that the immobilisation process enhanced the stability of laccase by increasing the activation energy (Ed), maintaining the ΔG° stability across temperatures, reducing the ΔH° for deactivation compared to the free enzyme, and decreasing the ΔS° due to decreased reaction system randomness [84]. The latter outcome results in an increased denaturation enthalpy (ΔH°), indicating greater stability due to a more rigid conformation, and a higher ΔG° for immobilised amylase, suggesting improved resistance to thermal unfolding at higher temperatures. Additionally, they found negative ΔS° values, which indicate that the immobilisation enhanced the orderliness of the enzyme structure, further enhancing its stability compared to the free enzyme [85].

Despite the importance of these parameters for optimising the enzyme’s performance in industrial applications and the proven success of immobilisation as a strategy for improved enzyme stability, the changes in thermodynamic parameters in immobilised enzymes remain poorly explored. Investigating these thermodynamic properties in immobilised enzymes would provide valuable insights into their enhanced stability and functionality, which would be crucial for their effective application in various beverage and food products or biotechnological processes.

### 3.5. Inhibition Resistance

The presence of substrates, products, or other components in the reaction medium can reduce the reaction rates and, in some cases, halt the reactions before reaching thermodynamic equilibrium. The immobilisation of enzymes, however, is an effective approach to mitigate these inhibition issues, depending on the specific inhibition mechanism used in a certain scenario [86]. Immobilisation can cause slight distortions in the enzyme’s active site when inhibitors interact with the protein, potentially reducing the impacts of the inhibition, especially for inhibitor binding sites more than for substrate binding sites. In cases of allosteric inhibition, where inhibitors bind away from the catalytic site, immobilisation can obstruct these sites, effectively circumventing the inhibition [9].

Enhancing the enzymatic activity by improving the resistance to inhibitors is a particularly relevant approach in beverage processing, such as for wine, beer, and other beverages. The use of immobilisation significantly improves the enzyme’s stability and functionality, making the enzyme more resistant against inhibitory compounds commonly found in these beverages. For instance, in winemaking, the enzyme’s activity is often inhibited by phenolic compounds, glucose, and ethanol. Immobilised enzymes demonstrate increased resistance to these inhibitors, maintaining activity over prolonged periods and under challenging conditions [41]. Similarly, in brewing, immobilised enzymes exhibit enhanced resistance to substances such as heavy metals, hop compounds, and ethanol, ensuring consistent performance during fermentation. Moreover, immobilisation mitigates product inhibition by glucose and galactose, preserving the enzymatic reactions in the production of lactose-free beverages [57].

For example, the activity of β-glucosidase is typically inhibited by glucose, limiting its industrial application. Its immobilisation on a monoaminoethyl–N-ethyl-agarose ionic support maintained high activity levels of 95% at glucose concentrations of 0.05–0.1 M and 50–75% at even higher concentrations. This biocatalyst also retained more than 80% of the initial activity within 24 h across all tested ethanol concentrations, suggesting protection against ethanol and glucose inhibition and enabling applications in ethanol-rich environments, such as wine [41].

In another case, β-galactosidase’s activity presented significant inhibition effects from glucose and galactose, with the soluble enzyme’s activity declining sharply under high galactose concentrations (1 M), whereas the immobilised β-galactosidase retained substantial activity (65%) due to structural changes that prevent inhibitor contact because of strong enzyme attachment to the matrix. The use of epoxy-activated Sepabeads led to decreased inhibition and enhanced enzyme stability even in denaturing environments [57].

Thus, the use of immobilisation results in more rigid structural conformation to enzymes, preserving the optimal active site configurations crucial for sustained catalytic activity. This rigidity reduces the likelihood of inhibitors binding to enzymes, thereby maintaining their enzymatic activity. Additionally, the immobilisation creates a microenvironment less susceptible to inhibitors, further protecting the enzyme’s functionality from inactivation in diverse industrial applications.

## 4. Enzyme Immobilisation to Enhance Enzyme Recovery and Reuse

Enzyme immobilisation systems offer a viable approach to improve the production yield and scale-up processes through the reuse of biocatalysts in batch processes [69].

The reuse of enzymes diminishes the production costs by maximising the use of catalytic compounds and minimises the waste generation and carbon emissions associated with enzyme manufacturing and disposal.

When immobilised, enzymes retain their catalytic activity and form insoluble biocatalysts that can be easily separated from the reaction medium through filtration, decantation, or magnetic separation. This reusability allows for multiple operational cycles, resulting in an increased production yield [9]. Studies have demonstrated significant improvements in product outputs, such as a 242% increase in the final product achieved by reusing immobilised pectinase in six hydrolysis batches [69]. Thus, enzyme immobilisation systems offer a sustainable and efficient solution for the food industry, offering enhanced productivity and optimised performance while mitigating the environmental impacts, as highlighted in Table 3.

Although some systems demonstrate good operational stability, generally, the enzymatic activity is lost as the immobilised enzyme is reused in a new hydrolysis cycle. Several factors can contribute to this loss, including mechanical damage during washing, enzyme release during incubation, protein denaturation due to temperature and hydrodynamic stresses, and changes in the carrier matrix’s structure due to repeated reuse and exposure to different temperatures and pH levels [44]. The accumulation of reaction products in the support’s internal microenvironment can also create mass transfer restrictions, resulting in enzyme inactivation [26,76]. Additionally, the repeated use of immobilised enzymes can weaken the binding strength between the matrix and the enzyme. The substrate’s interaction with the active site can cause distortion, reduce the catalytic efficiency, and lead to a loss of activity [87].

As observed in Table 3, the method chosen for immobilising enzymes is crucial in determining their reusability. Immobilisation systems obtained via physical adsorption, composed of weak attractive forces between the support and enzyme, as well as those using entrapment and encapsulation techniques, often result in low reusability in terms of the enzymatic activity retention and less cycles of reuse due to the risk of enzyme leaching from the immobilisation system. On the other hand, the covalent attachment method usually produces an immobilisation system with enhanced stability and reusability. In addition, the incorporation of cross-linking agents, including after the entrapment process, further improves the stability and reusability by preventing activity loss, swelling, disintegration, shape deformation, and enzyme leaching during the reuse cycles. Among the cross-linking compounds available, glutaraldehyde has been extensively researched and shown to be effective for multiple usage cycles in various studies [53,88,89,90,91].

**Table 3:** Operational stability of different systems of immobilised enzymes and substrates used for hydrolysis assays.

Enzyme and Source	Method	Carrier/Support	Substrate	Operational Stability	Ref.
Pectinase (*Aspergillus tamari*)	Adsorption	Zr-treated pumice	Pectin	72% enzymatic activity after 11 cycles of reuse.	[26]
Pectinase (*Penicillium oxalicum*)	Adsorption	Magnetic cornstarch microspheres	Pectin	60% enzymatic activity after 8 cycles of juice clarification.	[92]
α-Galactosidase (*Debaryomyces hansenii*)	Adsorption	Cellulose film	p-nitrophenyl α-D-galactopyranoside	100 and 80% enzymatic activity after 7 and 10 cycles of reuse, respectively.	[31]
β-Galactosidases (*Aspergillus oryzae*)	Adsorption	Chitosan beads	Fresh milk	80% enzymatic activity after 5 cycles of reuse.	[28]
β-Glucosidase (*Melaleuca pulchella*)	Adsorption	Monoaminoethyl–N- ethyl-agarose ionic support	p-nitrophenyl α-D-galactopyranoside	50% enzymatic activity after 20 cycles of reuse.	[41]
Pullulanase	Adsorption	Chitosan	Pullulan	70.8% enzymatic activity after 10 cycles of reuse.	[22]
Phytase (*Aspergillus niger*)	Adsorption	Zeolite modified with iron (II)	Soymilk	50% enzymatic activity after 6 cycles of hydrolysis.	[37]
Tannase (*Penicillium rolfsii*)	Entrapment	Calcium alginate beads	Pectin	Above 50% enzymatic activity after 6 cycles of reuse.	[21]
Tannase (*Escherichia coli*)	Entrapment + cross-linking	Calcium alginate beads cross-linked with glutaraldehyde	Propyl gallate	100, 80, and 40% enzymatic activity after 10, 26, and 46 cycles of reuse, respectively.	[90]
β-Glucosidase (commercial solution)	Entrapment	Sodium alginate gel beads	Cellobiose	96.5% enzymatic activity after 7 cycles of hydrolysis.	[79]
α-Galactosidase (*Aspergillus oryzae*)	Entrapment	Calcium alginate beads	Fresh milk	60% enzymatic activity after 5 cycles of reuse.	[28]
α-Acetolactate decarboxylase	Entrapment	Alginate gel beads	Z-Gly-Pro-pNA	Above 80% enzymatic activity after 6 cycles of reuse.	[27]
Naringinase (*Aspergillus niger*)	Entrapment	Calcium alginate beads	Naringin	Above 70% enzymatic activity after 7 cycles of reuse.	[64]
Pectinase (commercial preparation)	Covalent attachment	Polyaldehyde pullulan-activated glass beads	Apple pectin or galacturonic acid	80% enzymatic activity after 15 cycles of reuse.	[38]
Pectinase (*Aspergillus aculeatus*)	Covalent attachment	Dextran-aldehyde-cross-linked polyethyleneimine	Apple juice	Above 95% enzymatic activity after 10 cycles of reuse.	[93]
Pectinase (*Rhizopus* sp.)	Covalent attachment	Glutaraldehyde-activated bentonite	Pectin	Full and 87% enzymatic activity after 18 and 25 cycles of reuse, respectively.	[88]
Naringinase (*Penicillium decumbens*)	Covalent attachment	Glutaraldehyde-activated chitosan beads	Grapefruit juice	88.1% enzymatic activity after 10 cycles of reuse.	[48]
Naringinase (*Aspergillus niger*)	Covalent attachment	Dextran aldehyde-cross-linked magnetic polysaccharide carrier	Grapefruit juice	82.8% enzymatic activity after 10 cycles.	[94]
α-Galactosidase (*Citrullus vulgaris*)	Covalent attachment	Sepabeads EC-EP	p-nitrophenyl α-D-galactopyranoside	74 and 41% enzymatic activity after 18 and 30 cycles of reuse, respectively.	[95]
β-Galactosidase (*Bacillus circulans*)	Covalent attachment	Glutaraldehyde-activated chitosan beads	ONPG *	91% lactose conversion after 17 cycles of 165 min at 40 °C.	[53]
β-Galactosidase (*Kluyveromyces* sp.)	Covalent attachment	Magnetic cellulose	ONPG *	Hydrolytic efficiency of 50% after 15 cycles of reuse.	[96]
β-Galactosidase (*Aspergillus oryzae*)	Covalent attachment	Eupergit CM	Lactose	99.3% enzymatic activity after 20 cycles of reuse.	[20]
β-Galactosidase (*Kluyveromyces lactis*)	Covalent attachment	Glutaraldehyde-modified Immobead 150 supports (resin)	Cow’s milk	52% enzymatic activity after 15 cycles of reuse.	[97]
β-Galactosidase	Covalent attachment	Modified collagen supports	Cow’s milk	50% enzymatic activity after 17 cycles of reuse.	[71]
Papain (*Carica papaya*)	Covalent attachment	Poly(HEMA)–chitosan cryogels cross-linked with glutaraldehyde	Apple juice	86% enzymatic activity after 5 cycles of reuse.	[43]
Alkaline protease (*Bacillus licheniformis*)	Covalent attachment	Eupergit CM	Casein	Full enzymatic activity after 20 cycles of reuse.	[67]
Lactase (*Escherichia coli*)	Covalent attachment	Glutaraldehyde-activated magnetic nanocomposite	OPNG *	83.1% enzymatic activity after 20 cycles of reuse.	[34]
Laccase (*Trametes versicolor*)	Covalent attachment	Green coconut fiber activated with glyoxyl or glutaraldehyde	Apple juice	Above 80% enzymatic activity after 10 cycles of reuse.	[50]
α-Amylase (*Rhizoctonia solani*)	Covalent attachment	Chitosan beads cross-linked with glutaraldehyde	Soluble starch	80% enzymatic activity after 7 cycles of reuse.	[42]
β-Mannanase (*Aspergillus quadrilineatus*)	Covalent attachment	Glutaraldehyde-activated aluminum oxide pellets	Locust bean gum	Full enzymatic activity up to 10 cycles of hydrolysis.	[98]
β-Glucosidase (*Bacillus subtilis*)	Covalent attachment	Functionalised silicon oxide nanoparticles	Sugarcane juice	60% enzymatic activity after 10 cycles of reuse.	[74]
α-Acetolactate decarboxylase (*Brevibacillus brevis*)	Covalent attachment	Glutaraldehyde-activated chitosan beads	α-acetolactate	80% enzymatic activity after 12 cycles of reuse.	[63]
Xylanase (*Thermomyces lanuginosus*)	Covalent attachment	Trichlorotriazine-functionalised polyethylene-glycol-grafted magnetic nanoparticles	Pineapple juice	60% enzymatic activity after 9 cycles of hydrolysis.	[25]
Xylanase (*Trichoderma longibrachiatum*)	Covalent attachment	Glutaraldehyde-activated silica gel supports	Orange juice	77% enzymatic activity after 10 cycles of reuse.	[36]
Pectinase (commercial preparation)	Covalent attachment	Genipin-activated chitosan particles	Orange/grape juice	50 and 40% relative clarification after 10 cycles for the orange juice and grape juice, respectively.	[99]

* ONPG: o-nitrophenyl β-d-galactopyranoside; Z-Gly-Pro-pNA: N-CBZ-glycyl-L-proline 4-nitroanilide.

Shafi et al. [100] compared immobilisation systems for β-galactosidase (*Aspergillus oryzae*) on a glutaraldehyde–polyaniline-doped magnetic graphene nanocomposite using both covalent attachment and adsorption (without glutaraldehyde). After ten reuse cycles, the covalent attachment method retained 91% of the initial enzymatic activity, while the adsorption method retained only 74%. Another study also evaluated the operational stability of xylanase immobilised through covalent attachment using glutaraldehyde or carboxy-propyl-activated silica gel supports and via adsorption using propylsulfonic acid or aminopropyl-functionalised silica gel [36]. After ten reuse cycles, the covalent attachment systems maintained more than 70% of the residual enzymatic activity. In contrast, the adsorption methods retained less than 15% of the enzymatic activity in the 10th hydrolysis cycle [36].

Further studies provide evidence of the limited reusability and lower enzymatic activity levels in enzymes immobilised through entrapment. Most of these studies evaluated up to ten hydrolysis cycles, with examples including β-glucosidase [79], retaining a maximum of 96.5% enzymatic activity after seven cycles, and tannase [21], retaining 50% catalytic activity after six cycles. However, incorporating a cross-linking agent post-entrapment significantly enhanced the enzymatic activity retention and reusability. *E. coli* tannase was entrapped in calcium alginate beads and cross-linked with glutaraldehyde afterwards [90]. The authors achieved efficient enzyme utilisation rates for 46 hydrolysis cycles, with 80% and 40% of the initial enzymatic activity remaining after 26 and 46 reuse cycles, respectively. Similarly, Su et al. [101] observed 86.9% residual activity after 30 hydrolysis cycles by applying glutaraldehyde post-entrapment to tannase in sodium alginate beads. These studies demonstrated that the cross-linking agents were decisive for maintaining high enzymatic activity levels compared to immobilisation systems relying solely on physical adsorption or entrapment.

Numerous studies have explored the reusability of immobilised enzymes and reported varying outcomes depending on the substrate. For example, Yavaser and Karagozler [43] examined the immobilisation of papain on poly(HEMA)–chitosan cryogels activated with glutaraldehyde. They found that the enzymatic activity remained relatively stable over multiple cycles of proteolysis when using casein or apple juice as the substrate. Gennari et al. [102] observed better retention of catalytic activity for immobilised β-galactosidases on glutaraldehyde-modified Immobead 150 supports when working with cheese whey and milk compared to lactose solutions. Variations in activity retention levels were also observed depending on the substrate’s composition and concentration while studying the performance of immobilised phytase on zeolite modified with iron (II) [37]. Additionally, Dal Magro et al. [103] noted differences in the operational stability of immobilised pectinase when applied to various juices, potentially influenced by the presence of polyphenols, proteins, and starch in the beverages. These findings suggest that the substrate’s nature, including its composition and specific components, can influence the stability and reusability of immobilised enzymes by affecting factors such as the diffusional aspects and creating a favourable environment for enzymatic activity. Some components can act as stabilisers, protecting the catalytic compound from deactivation and influencing the diffusion of the substrate and product.

## 5. Enzyme Immobilisation to Facilitate Continuous Reaction Treatment

Continuous processing technology is a process that operates based on continuous flow, with no interruption, as opposed to batch processing. It has gained popularity in beverage production due to its numerous advantages over traditional batch processing methods. One of the primary benefits is the significant increase in efficiency, leading to higher production capacity and output levels, reduced costs [104], and consistent product quality by preserving the sensory characteristics of the beverage, such as the taste, aroma, and appearance. In addition, continuous processes are highly adaptable, allowing for rapid adjustments of the production parameters to meet new demands and product specifications [105]. Enzyme immobilisation systems, in conjunction with continuous processing technology, offer additional benefits. The use of immobilised enzymes can improve the product purity by reducing enzyme contamination. This also prevents the enzymatic activity from being impacted or stopped by the product accumulation, which may inhibit the catalytic activity. Moreover, this can lead to improved quality control and enable the isolation and reuse of enzymes from the reaction broth.

**Figure 4:**
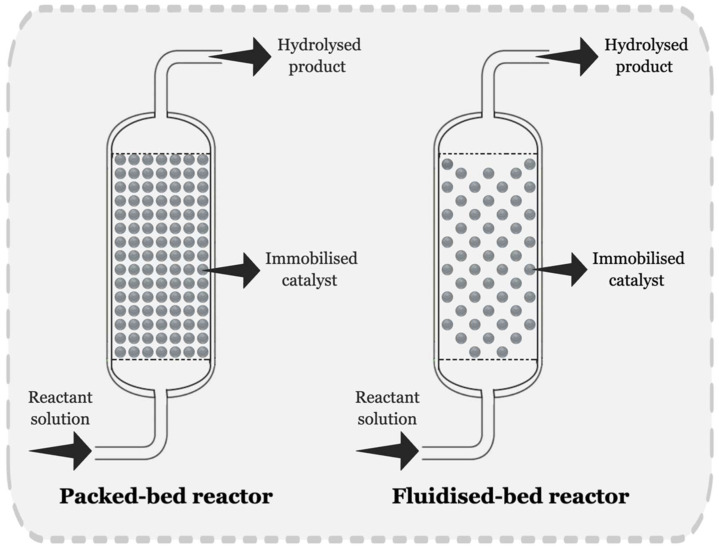
Diagram of packed-bed and fluidised-bed reactors.

Various reactor configurations can facilitate catalytic reactions through the use of immobilised enzymes. Among them, packed-bed and fluidised-bed reactors are commonly employed and explored in beverage processing for continuous operation [6,19,106,107,108]. Figure 4 shows a diagram of packed-bed and fluidised-bed reactors. Packed-bed reactors contain immobilised enzymes within a fixed bed, through which the solution is pumped. Their large surface area, low operational cost and reduced damage to the biocatalyst, due to less shear stress, make them an attractive option for industrial applications. However, bed compaction and the formation of preferential paths can limit mass and heat transfer efficiency. On the other hand, fluidised-bed reactors maintain immobilised biocatalysts in suspensions by circulating air or the substrate solution through the system (Figure 4), thereby avoiding the decantation of immobilised enzymes because the inlet for the substrate is positioned at the bottom of the reactor [6]. The fluidised-bed reactor design effectively removes preferential paths and prevents column clogging by allowing suspended matter to flow through the space between immobilised biocatalysts. This feature facilitates mass transfer and ensures a better distribution of the biocatalyst throughout the column [109]. However, it can cause damage to the immobilised enzyme and accommodates the use of smaller amounts of enzyme biocatalyst per unit volume than packed-bed reactors. As a result, it reduces the reactor’s efficiency and makes scale-up more challenging [6].

Both reactor configurations possess inherent advantages and disadvantages and have been extensively used and evaluated in scientific studies. Fluidised-bed reactors have been less explored in the literature. Girigowda and Mulimani [55], for example, entrapped α-galactosidase from *Aspergillus oryzae* into glutaraldehyde–K-carrageenan beads and achieved 92% removal of non-digestible oligosaccharides in soymilk. Similarly, entrapped pectinase (*Aspergillus aculeatus*) used to clarify apple juice in a fluidised-bed reactor achieved a substrate conversion rate of 97.2% [19]. Continuous treatment in a fluidised-bed reactor was also studied using immobilised prolyl-endopeptidase to achieve a reduction in the amount of intact gluten in beer. The continuous treatment reduced the initial gluten content (65 mg/kg) in the commercial beer from barley malt to a concentration of 15 mg/kg after 10 h of treatment [110]. Pomegranate juice was also treated in a fluidised-bed reaction using a multi-enzymatic system containing protease, polygalacturonase, and pectin lyase. The authors observed that samples enzymatically treated with the continuous system presented better native phenolic pattern retention ability, showing higher contents of both total and monomeric anthocyanins compared to the untreated juice [111]. In contrast, the packed-bed reactor approach has been widely researched and used in various industrial applications combined with the covalent attachment immobilisation of enzymes. Such systems have been employed to accomplish high hydrolysis performance levels in milk and for various types of fruit juice clarification and debittering processes [19,106,107,108].

The careful selection of an appropriate reactor and enzyme immobilisation system is essential to ensure optimal performance and maximum substrate conversion rates. The entrapment and encapsulation of enzymes, for instance, may lead to increased diffusional limitations, since the catalytic compounds are primarily contained within the immobilisation matrix. In this regard, the use of a fluidised-bed reactor may provide a more suitable option by facilitating mass transfer through substrate circulation. Additionally, most catalytic compounds are protected within the immobilisation system, thereby reducing enzyme losses from structural damage, disintegration, or leaching. On the other hand, the use of enzymes immobilised on the surface is deemed more suitable for packed-bed reactors owing to the limitations associated with mass and heat transfer and the lower risk of impairment to the immobilised biocatalyst. Despite some activity loss, using a cross-linking agent alongside the correct type of reactor optimises the process and enables the reuse of immobilised enzymes without significantly compromising their hydrolysis performance.

Reutilising immobilised enzyme systems after beverage production or removing the immobilised catalytic compound for equipment cleaning can further enhance the production yield when using the same catalyst. The catalytic activity is expected to decrease due to enzyme inactivation or leaching from the matrix. Hence, to address this issue, different strategies have been employed. Patil et al. [112], for instance, used glutaraldehyde and boric acid for immobilisation. The cross-linking agent facilitated superior enzymatic retention, improved stability, and catalytic activity rates, guaranteeing minimal enzyme leakage during six subsequent uses of the immobilised α-galactosidase system applied in a continuous process. The hydrolysis conversion rate remained high for up to six cycles, with an average decrease of 3.6% in the conversion of lactose after each cycle. Likewise, González-Temiño et al. [113] confirmed that a cross-linking agent enhances the stability of the immobilisation system and performance of the enzymatic membrane bioreactor. Without a cross-linking agent, approximately 39.5% of the immobilised naringinase was desorbed, rapidly decreasing the naringin conversion rate. However, only 7.3% of the naringinase was released after cross-linking, indicating the presence of more stable systems and the possibility of reuse. In addition, no apparent decrease in conversion of the bitter compound was observed in the degradation of naringin in grapefruit juice during three hydrolysis cycles.

Another strategy to overcome reaction rate reductions due to enzyme leaching is adjusting the flow rate. Fidaleo and Tavilli [114], for example, adjusted the flow rate after each cycle to maintain a consistent reaction rate and optimise the conversion of urea to ammonia. This adjustment compensated for any biocatalytic activity or stability changes over repeated cycles and ensured the system operated under similar conditions for each cycle.

The impact of the flow rate on the hydrolysis efficiency is substantial, as evidenced by Fidaleo and Tavilli’s [114] study and the experimental data presented in Table 4. The results demonstrate a negative correlation between an increased flow rate and substrate-to-product conversion in packed-bed and fluidised-bed reactors. This effect can be attributed to a reduction in the residence time of the substrate solution within the reactor, which limits its interaction with the biocatalytic compound. At lower flow rates, diffusional transport of the substrate to the inner regions of the immobilised biocatalyst is facilitated, promoting effective hydrolysis. Conversely, a faster flow rate may not allow sufficient time for enzymes to catalyse the reaction or for the substrate to penetrate the biocatalyst particles, leading to a decrease in hydrolysis capacity [114]. Along with the short residence times, packed-bed reactors may also experience particle accumulation, which increases the diffusional challenge because of the presence of dead zones and preferential pathways for the substrate [6,104]. The approaches for mitigating these problems include increasing the residence time, which can be problematic. A prolonged residence time can impact the enzyme’s stability, activity, and microbial contamination [104]. Thus, precise control of the residence time is crucial in designing and implementing an effective and continuous enzymatic process in a reactor. The residence time regulates the interaction time between the substrate and enzyme, which ensures better substrate–product conversion. Consequently, the use of the optimal residence time can lead to enhanced process efficiency, minimised reaction times, and reduced costs [115].

## 6. Enzyme Immobilisation and Its Application in the Beverage Sector

Enzymes are indispensable in the beverage processing sector. They efficiently transform raw materials into products with desirable flavours, aromas, and textures [5]. They offer innovative alternatives to chemical or mechanical methods, enhancing the yield and quality across the beverage industry. From beer and wine production to juice manufacturing and beyond, enzymes play a crucial role in viscosity and turbidity control, colour and flavour extraction, protein stabilisation, nutritional and texture improvements, and bitterness reductions [3]. The industrial application of enzymes is further enhanced by the use of immobilisation systems, as detailed below.

### 6.1. Application in Winemaking

In winemaking, enzymes are commonly used to generate a series of flavour compounds, such as terpenes, pyrazines, phenols, and esters [5], enhancing the product quality and speeding up the ageing process. Immobilised glycosidases, prepared through aggregation and cross-linking, have been used to control aroma development while extending the enzymatic lifecycle, facilitating reuse, and allowing enzyme removal from wine [122]. Studies have shown noteworthy aroma improvements with the use of immobilised glycosidases [49,123,124]. For instance, a significant increase in free monoterpenes (3.4-fold over the flavour threshold) was observed after 20 days of treatment of muscat wine with immobilised enzymes (β-glucosidase, α-arabinosidase, and α-rhamnosidase from a commercial preparation), resulting in a more intense fruity and floral aroma [123]. Along with the production of desirable aroma compounds, the immobilisation of commercial β-glucosidase onto chitosan beads required a substantially reduced enzyme dosage (367 times less than for the free enzyme), while maintaining stability and enabling reuse across different batches [49]. Similar findings were observed when immobilising β-D-glucosidase and α-L-arabinofuranosidase in chitosan beads to hydrolyse glycosides in white wine [124]. The immobilisation process also ensured the retention of the enzymatic activity for an extended period (up to 91 days of incubation under winemaking conditions). Additionally, the immobilised enzyme beads could be easily removed via filtration at any stage of the winemaking process, enabling better control over the reaction and achieving the desired sensory attributes while reducing the need for further purification steps [124].

Along with flavour and aroma enhancements, other applications of immobilised enzymes in winemaking have included correcting excessive colour production issues during the prolonged maceration process [41], preventing protein haze formation [66,121,125,126,127], producing lower-alcohol content wines [30,128], and histamine removal [35]. Enzymatic treatment has emerged as a solution for colour removal, which represents a potential opportunity for producing lighter-coloured wines such as rosé wines from red grape varieties [41]. For example, immobilised β-glucosidase was employed for the clarification of wine and grape juice by hydrolysing anthocyanin [88]. The authors observed that the immobilised enzymes remained stable even in the presence of up to 0.1 M glucose for 24 h of incubation, and retained up to 70% of the initial enzymatic activity with ethanol concentrations of 5, 10, and 15% [88]. Immobilised enzymes were also used to prevent protein haze formation, a key instability issue of non-microbial origin, during white wine storage. While bentonite clay is a fining agent commonly used for stabilising white wines, the random binding of proteins can negatively impact the wine quality [129]. Consequently, researchers have explored the efficacy of using immobilised proteolytic enzymes as an alternative to bentonite fining and presented an immobilisation system for enzymes as a promising technology for reducing protein haze in white wine [66,125,126,127]. Papain (*Carica papaya*) immobilised on commercial chitosan beads resulted in protein haze reductions ranging from 14 to 68% in a continuous packed-bed reactor treatment [121]. The potential use of immobilised enzymes as a biotechnological option for producing lower-alcohol content wines was also explored. Studies have immobilised glucose oxidase [30] and enzyme extracts from *Geotrichum* spp. strains [130] in calcium alginate beads to solve the problem of higher sugar concentrations in ripened grapes, which implies a higher alcohol content in wine (exceeding 15%) and results in undesired and unpalatable aromas and flavours. Glucose oxidase and glucose catalase co-immobilised in a silica–calcium–alginate hydrogel were also applied to reduce the alcohol content by up to 37.3 g/L of glucose in treated must, corresponding to a decrease in potential alcohol strength of 2.0% volume [128].

Lastly, a study investigated the removal of histamine using glyceraldehyde-3-phosphate dehydrogenase immobilised on magnetic nanoparticles. Wang et al. [131] presented a histamine degradation rate of over 80%, with minimal impact on the wine’s composition, improved pH tolerance and thermostability, and excellent reusability (nearly 60% activity retained after five reuse cycles) compared to free enzymes.

### 6.2. Application in Brewing

In beer fermentation, diacetyl is a by-product that can cause an unpleasant buttery taste, even at low concentrations such as 0.15 ppm. To prevent this problem, the industry usually uses α-acetolactate decarboxylase during the beer fermentation process to stop diacetyl formation and shorten the maturation period [27]. Researchers evaluated α-acetolactate decarboxylase entrapped in alginate microbeads [27]. The immobilisation system exhibited great potential for brewing applications. It reduced the diacetyl content to below 0.1 ppm in just 7 days, faster than for the soluble enzyme. The immobilised enzymes also sped up the beer maturation process, were easily recycled via simple filtration, and retained around 80% of their initial activity after six cycles of use [27]. The enzymatic activity of immobilised α-acetolactate decarboxylase on glutaraldehyde-activated chitosan beads was also investigated to prevent off flavours in beer [63]. This immobilisation method stopped possible conformational changes during pH shifts and preserved 90% of the enzymatic activity at pH 5.0 compared to a 50% reduction for free enzymes. The immobilised enzymes also showed better stability in the presence of alcohol, retaining 98% of their activity compared to 80% for free enzymes. In addition, 80% of their enzymatic activity was retained after 12 reaction cycles [63].

Beer, a complex mixture of many components, has relatively weak stability levels, resulting in turbidity and precipitation during storage [22]. Chill haze, formed by sensitive protein and polyphenol non-covalent bonds, is a common concern in the brewing industry. Most protein–polyphenol complexes are removed through precipitation by cooling the fermentation liquid during beer maturation and a subsequent clarification process using silica gel or polyvinylpolypyrrolidone. However, both clarification methods can reduce the natural antioxidants and limit the protein-absorbing capacity [132]. To address this problem, researchers investigated the immobilisation of proline-specific endo-protease on non-porous silica nanoparticles [132]. The immobilised enzymes effectively prevented turbidity in treated refrigerated beer. After refrigeration, the turbidity of the untreated beer increased from 0.6 to 3.1, while the immobilised enzymes and silica-gel-treated beer presented no obvious cold muddy phenomenon. A small increase in turbidity was measured compared to the blank sample, proving that the cold turbidity protein was efficiently removed by both methods and the immobilised enzyme approach could easily replace the traditional method [132].

Another study explored the use of pullulanase immobilised on chitosan beads to produce a modified membrane for one-step beer refining and to lower the alcohol–ester ratio for efficient biological ageing [22]. Besides improving the beer flavour, the modified membrane effectively filtered out the protein and β-glucan, which are responsible for causing turbidity in beer, while retaining beneficial amino acids and vitamins in the beverage. Moreover, due to the presence of chitosan, the modified membrane significantly inhibited bacterial growth (*Escherichia coli* and *Staphylococcus aureus)*, extending the shelf life. Furthermore, the immobilisation process improved the enzymes’ thermal, pH, organic solvent, and storage stability rates. It also demonstrated excellent performance after ten reaction cycles, retaining up to 70% of the enzymatic activity [22].

Another promising and interesting application of immobilised enzymes in the brewing process is the production of reduced-gluten beer through a continuous fluidised-bed treatment with the application of immobilised prolyl endopeptidase. After immobilising the protease on chitosan beads, the optimum temperature was broadened to a range of 50–60 °C, and the optimal pH remained in the typical range for beer (4.2 to 4.5). Moreover, the reduced gluten content in the commercial beer made from barley malt was under the minimum requested for gluten-free products (<20 mg/kg) [110].

### 6.3. Application in Vegetable and Fruit Juice Production

Crushed pectin-rich fruits result in gelatinous and high-viscosity juices and fruit pulps with increased turbidity [98]. The turbidity or cloudiness is caused by the colloidal dispersion of polysaccharide components such as pectin, cellulose, and hemicellulose in the fruit juices. This not only compromises the product quality during processing and storage but also negatively impacts the level of consumer acceptance [25]. Several enzymes, including pectinase, laccase, xylanase, poly-galacturonase, β-glucosidase, and pectin methylesterase, have been explored for reducing turbidity in juices and increasing production yields, as summarised in Table 5. Many studies comparing free and immobilised enzymes have shown significant reductions in turbidity and viscosity. Such improvements can be attributed to the enhanced stability of the immobilised enzymes, leading to better catalytic performance under the harsh conditions characteristic of fruit juice, such as low pH levels.

In addition to the reductions in turbidity and viscosity, da Silva et al. [133] observed relatively minor variations in the total soluble solid contents, reducing sugar contents, and pH values in juices when using chitosan–pectinase or silica–pectinase immobilisation systems for orange juice clarification. They also noticed that the optimised clarification process significantly enhanced the colour of the orange juice due to the turbidity reduction, maintaining the desired vibrant and vivid yellow appearance. Proteases have also been explored for fruit juice clarification in the literature. Yavaser and Karagozler [43] developed a one-step clarification process using immobilised protease to prevent interactions between polyphenols and haze-active proteins. While gelatin and silica are widely applied to block polyphenol–protein complex formation, these materials have drawbacks, such as reducing the polyphenol levels, requiring long operation periods, and involving vigorous filtration processes. However, Yavaser and Karagozler [43], through their one-step clarification process, observed a depletion in the total phenolic content of only 8.2%. In another study, a multi-enzymatic immobilisation system containing both protease and pectinase on functionalised chitosan beads was developed and applied for pomegranate juice clarification. The system resulted in immediate and potential turbidity depletion rates of 49 and 70%, respectively, after 21 days. In addition to the significant reduction in haze-active molecules, the enzymatically treated juices better preserved the anthocyanin pattern compared to the untreated juice over time [111].

**Table 5:** Studies performed using immobilised enzymes for juice clarification to reduce the turbidity and viscosity.

Enzyme/Source	Method	Carrier/Support	Turbidity Reduction (%)		Viscosity Reduction (%)		Operational Stability	Juice Type	Ref.
			FE	IE	FE	IE			
Tannase (*Penicillium rolfsii*)	Entrapment	Calcium alginate beads	73.0	78.0	No change	44.0	Above 50% activity after 6 reuse cycles.	Apple	[21]
Polygalacturonase (*Aspergillus niger*)	Physical adsorption	Calcium alginate beads	94.5	96.8	-	-	20% activity after 10 reuse cycles.	Apple	[73]
Pectin Lyase (*Acinetobacter calcoaceticus*)	Covalent attachment	Magnetic carboxymethyl cellulose nanoparticles	49.2	54.4	12.5	28.6	-	Plum	[65]
Pectinmethylesterase (*Lycopersicon esculentum*)	Entrapment	Calcium alginate beads	-	98.0	-	55.0	55% activity after 10 reuse cycles.	Orange	[60]
Alkyne-pectinase (*Aspergillus aculeatus*)	Covalent attachment	Polyethyleneimine cryogel support	100.0	55.0	-	-	61% activity after 12 reuse cycles.	Apple	[80]
Pectinase (*Aspergillus aculeatus*)	Cross-linking	Epoxy polymer support	99.5	99.5	27.9	27.8	Above 95% activity after 10 reuse cycles.	Apple	[93]
Exopolygalacturonase (*Penicillium paxilli*)	Covalent attachment	Chitosan magnetic nanosupport	93.0	93.6	51.6	55.0	63% activity after 4 reuse cycles.	Grape	[33]
Xylanase (*Thermomyces lanuginosus*)	Covalent attachment	Magnetic polyethylene trichlorotriazine nanoparticles	52.8	42.0	-	-	50% activity after 9 reuse cycles.	Pineapple	[25]
Pectinase (*Aspergillus aculeatus*)	Covalent attachment	Cross-linked alginate–montmorillonite beads	77.0	80.50	39.10	40.0	53% activity after 6 reuse cycles.	Pineapple	[29]
Pectinase (*Aspergillus aculeatus*)	Covalent attachment	Magnetic polyethylene trichlorotriazine nanoparticles	48.0	59.0	-	-	60% activity after 9 reuse cycles.	Pineapple	[75]
Xylanase (*Aspergillus flavus*)	Entrapment	Calcium alginate beads	51.6	52.8	5.2	17.8	63% and 22% activity after 8 and 12 reuse cycles, respectively.	Pineapple	[61]
Pectinase (*Aspergillus aculeatus*)	Entrapment	Calcium alginate beads	-	97.2	-	20.8	80% and 30% activity after 3 and 8 reuse cycles, respectively.	Apple	[19]
Xylanase (*Trichoderma longibrachiatum*)	Covalent attachment	Activated silica gel supports	74.7	73.6	-	-	77% activity after 10 reuse cycles.	Orange	[36]
Pectinase (*Rhizopus* sp.)	Covalent attachment	Activated bentonite clay	-	61.6	-	63.9	87% activity after 25 reuse cycles	Orange	[88]
Pectinase (*Aspergillus niger*)	Entrapment	Synthetic polyvinyl alcohol sponge	Hazed sample	Cleared sample	69	75	97.5% and 91% activity after 10 and 12 reuse cycles, respectively.	Orange	[134]
Xylanase (*Bacillus pumilus*)	Covalent attachment	Activated aluminum oxide pellets	1.0	2.0	80.0	79.8	55% activity after 5 reuse cycles.	Papaya	[45]
Xylanase (*Bacillus pumilus*)	Covalent attachment	Activated aluminium oxide pellets	27.0	30.0	35.0	60.0	85% and 58% activity after 5 and 10 reuse cycles, respectively.	Grape	[39]
Pectinase (Pectinex^®^ Ultra Color)	Cross-linking	Glass beads	33.3	39.8	96.5	96.5	80% activity after 15 reuse cycles.	Barberry	[72]
Pectinase *(Penicillium crustosum*)	Covalent attachment	Amino-functionalised magnetic core–shell nanoparticles	64.0	62.0	-	-	85% activity after 5 reuse cycles	Orange	[135]

Immobilised enzymes such as naringinase were also employed to reduce the bitterness of juices [99,100,101,102] and proved superior to the classic resin treatment at effectively reducing the bitterness while maintaining the levels of other bioactive compounds [64]. A comparative study of the effect of debittering pomelo juice using immobilised naringinase and resin on the physicochemical and phytochemical properties was performed. The enzyme-treated juice retained higher percentages of the physicochemical and bioactive compounds, presenting minor impacts on the soluble solid content, acidity, ascorbic acid content, and phenolic content compared to the resin-treated juice, besides having an efficacious debittering effect [64]. Another study also observed a positive impact on the debittering process by using immobilised naringinase [113], whereby 73% of the naringin was hydrolysed by immobilised naringinase on polyethersulfone ultrafiltration membranes. The enzymatic membrane was also reutilised for the debittering of grapefruit juice over at least three cycles, achieving a 50% reduction in naringin content without modifying the pH, soluble solid content, or titratable acidity of the juice, presenting a minimal reduction in the antioxidant capacity [113]. A bitterness reduction was also achieved by converting free limonoids into glucosides using glucosyltransferase, which was immobilised on different carriers such as chitosan cross-linked with glutaraldehyde, cellulose carbonate, and PVC [136]. A taste improvement was also achieved by immobilising fungal cellulase on a xerogel matrix, resulting in increased saccharification and volume yields [137].

Immobilised enzymes have found further applications in reducing the patulin content, a toxic compound produced by moulds in fruits, which poses significant health risks. Enzymes of *Pseudomonas aeruginosa* with patulin degradation ability were entrapped in calcium alginate beads and effectively reduced the patulin levels in apple juice by 95% within 96 h [138]. Cellulose-based magnetic nanomaterial-immobilised esterase was also used as an effective detoxification agent for patulin in apple juice. The detoxification rate of the patulin exceeded 80%. The immobilisation process also enabled the rapid separation and recovery of the enzyme, allowing 8 usage cycles and retaining approximately 50% of the initial enzymatic activity [139]. The enzymatic reactions resulted in non-cytotoxic products, demonstrating that the new detoxification method holds promise for enzymatic applications in mitigating mycotoxin contamination without compromising the quality of the fruit juice.

Lastly, enzymes were immobilised to convert high-calorie fruit juices, such as mango, orange, and sugar cane juices, into low-calorie rare sugars, potentially serving as a sucrose substitute for people with diabetes. D-allulose-3-epimerase was immobilised onto an epoxy support as a reusable biocatalyst to efficiently produce D-allulose from D-fructose. This method presents a viable approach for the industrial production of functional fruit juices, meeting the increasing demand for functional and health-conscious products [140].

### 6.4. Application in Dairy Beverages

Bovine milk is a complete nutritional food and is considered by many to be one of the best substitutes for human milk. Certain proteins found in cow’s milk, such as caseins, β-lactoglobulin, and α-lactalbumin, can trigger allergic reactions in infants and young children, affecting their growth and development and even posing health risks [141]. Partially or extensively hydrolysed milk formulas have been developed as alternatives for infants to prevent allergic reactions while preserving the essential amino acids for growth. Enzymatic hydrolysis is a popular and widely used technique for reducing protein allergenicity, thanks to its remarkable efficiency and substrate specificity under mild conditions [142]. The specificity of the protease enzyme determines where the protein is broken down, influencing the hypo-allergenicity of the resulting hydrolysate. However, enzyme inactivation via heating can alter the antigenicity of the hydrolysate. Therefore, immobilising the enzymes could overcome this issue [143]. Although the use of protease immobilisation systems has been extensively studied, the research on their use for reduced allergenicity is still limited. For example, the use of immobilised papain on polyacrylamide hydrogel microspheres was evaluated [142] and the study found that the immobilisation did not alter the optimal pH or temperature but rather significantly improved the pH and temperature stability. The immobilised enzyme significantly hydrolysed β-lactoglobulin and α-lactalbumin within 10 min and achieved a significant reduction in these allergenic proteins within 30 min. In addition, the immobilised papain was successfully reused for three complete protein hydrolysis cycles [142]. Similarly, alcalase and neutrase were immobilised onto glutaraldehyde-activated amino-modified magnetic nanoparticles [143]. This system showed comparable results to free enzymes and produced milk protein hydrolysates that met the requirements for an extensively hydrolysed formula with reduced antigenicity. Studies focusing on immobilising alkaline protease from *Bacillus licheniformis* [67] and trypsin [144] demonstrated that the enzymatic properties were not compromised after immobilisation. Therefore, further research and development of protease immobilisation systems hold promise as an effective alternative approach for protein hydrolysis and the production of allergen-free products.

Enzyme immobilisation systems have found other valuable applications in the dairy beverages industry, specifically in the hydrolysis of lactose. Immobilised β-galactosidase enzymes have been extensively studied with various types of bovine milk (whole, skimmed, powder, fresh, and UHT milks) through batch and continuous processing. This enzymatic process aims to remove lactose, making dairy products suitable for individuals with lactose intolerance. Beyond lactose hydrolysis, these immobilisation systems have several other applications within the dairy processing industry. The immobilisation of β-galactosidase can be employed to produce energy supplements from milk and cheese whey, as well as to extract valuable prebiotic sugars from milk processing by-products. Additionally, immobilised β-galactosidases are used to treat dairy waste before whey disposal in water treatment plants [96]. Various natural polymers and inorganic materials have been investigated for immobilising β-galactosidase, including cellulose [109,131,145], chitosan [24,28,53,54,62,146,147,148], alginate [28], collagen [71], agarose [149], silica [147], glass beads [108,150], magnetic supports [34,96,151,152], and others [62,87,97].

Wolf et al. [153], for instance, developed a chitosan-hydrogel-based immobilisation system for β-galactosidase. After ten cycles of lactose hydrolysis in UHT milk, the immobilised lactase retained 71.8% of its enzymatic activity. Similarly, Jin, Li, Ren, and Lee [151] also achieved impressive results with polyaniline-nanofiber-immobilised β-galactosidase, maintaining 98% catalytic function after ten cycles of lactose hydrolysis and producing a similar amount of glucose as for the first use. The immobilisation system exhibited high stability over a wide pH range, and after 12 days of incubation at 40 °C, the enzymatic activity retention rate was 96%. In another study, it was found that β-galactosidase immobilised on magnetic cellulose had 1.2 times higher substrate affinity than the free enzyme. The immobilisation process also increased the thermal stability by up to seven-fold, with 50% relative enzymatic activity retained after fifteen hydrolysis cycles in milk [96].

### 6.5. Application in the Production of Plant-Based Dairy Alternatives

Plant-based beverages, including those made from cereals, pseudo-cereals, legumes, and seeds, have become popular dairy alternatives for individuals with lactose intolerance, dairy allergies, or cholesterol issues or those opting for a plant-based diet. These beverages are typically made from coconut, almond, cashew, soy, rice, barley, oat, and other sources, which may contain compounds that can cause digestive discomfort [154]. Such compounds include soluble raffinose and stachyose, which are anti-nutrient compounds known for causing flatulence and abdominal discomfort [47]. To improve the consumer acceptance and enhance the nutritional value of plant-based beverages, researchers have focused on removing these indigestible compounds. One effective method involves immobilising α-galactosidases, enzymes that hydrolyse the raffinose family oligosaccharides (RFOs) found in soymilk, for example. Studies have shown that immobilising α-galactosidases using different matrices such as calcium alginate [47,52,59], gelatin [155], chitosan [47], and Sepabeads [95] significantly reduces the RFO content. In one study, chitosan and calcium alginate beads were used to immobilise α-galactosidases. Significant reductions in RFOs of 97.6% and 93.7%, respectively, were found after 4 h of hydrolysis [26]. This was much more effective than for the free enzyme, which only hydrolysed 30% of the RFOs under the same conditions. Another study by Çelem and Önal [95] showed the outstanding storage stability of α-galactosidase immobilised on Sepabeads, retaining 78% of the initial activity after eight months and removing 74% of the raffinose after 18 hydrolysis cycles. The immobilisation of α-galactosidase on a cellulose film also showed excellent stability and enzymatic activity, suggesting its potential use as an active packaging option for soymilk to reduce the RFO content during storage [31].

Phytic acid is another anti-nutrient found in seeds and cereal grains. It is the primary storage form of phosphate in these food sources, and can adversely affect human health and nutrition by binding to essential minerals such as calcium, zinc, and iron, forming mineral–phytic acid complexes. This complex formation can lead to deficiencies in these nutrients, causing conditions such as anaemia, hypocalcaemia, and other related health issues, especially in populations consuming plant-based diets [156,157]. Consequently, various immobilisation systems for phytase, including the use of starch agar beads [156], zeolite modified with iron II [37], chitosan activated with glutaraldehyde [158], cellulose beads [157], calcium alginate beads [158], Sepabeads [159], and glass microspheres [157], have been developed. Systems utilising phytase immobilised on chitosan beads and entrapped in calcium alginate beads preserved the original enzymatic activity for 8 and 6 cycles of reuse, respectively [158], while Ushasree, Gunasekaran, and Pandey [156] achieved a 10% increase in the release of inorganic phosphate relative to the free enzyme in soymilk.

The enrichment of isoflavone aglycones in black soymilk has also been studied through the use of immobilised enzymes to remove the glucoside conjugates via β-glucosidase action. Isoflavones, a subgroup of flavonoids, possess health benefits such as cancer, osteoporosis, and cardiovascular disease prevention and antioxidant effects [160]. The conversion of isoflavone glucosides into aglycones is desirable for faster absorption and enhanced bioavailability [161]. Hence, studies have investigated the use of immobilised β-glucosidase using various carriers such as chitosan [162], cellulose beads [160], spent coffee grounds [51], glass microspheres [163], nylon pellets [163], and PAN beads [163] to enrich the isoflavone aglycones in black soymilk. Chen, Yao, Chen, Lo, Yu, and Cheng [160], for example, achieved the complete deglycosylation of daidzin and genistin isoflavones, enriching the black soymilk with two isoflavone aglycones by 67% in just 30 min of hydrolysis using a β-glucosidase–cellulose immobilisation system. The immobilised enzyme maintained 70% of its original activity over ten consecutive uses and remained stable for ten days in black soymilk, providing an economically viable approach for industrial production instead of fermentation. Another study by de Ávila et al. [164] reported significant increases in isoflavone aglycone content (by 36–46 times) after using free and immobilised tannase forms in soymilk.

Other enzyme immobilisation systems have been investigated for plant-based beverage applications. Neta et al. [165] focused on improving the stability of coconut milk using lipase immobilised on acrylic resin and chitosan. They synthesised sugar esters such as fructose, sucrose, and lactose esters, which effectively reduced the surface tension of the fresh coconut milk to stabilise the emulsion. Sahoo et al. [166] used immobilised proteases in calcium alginate beads to extend the shelf-life of soymilk by up to 15 days while improving its aroma and reducing the beany flavour. Patil, Kote, and Mulimani [112] targeted the removal of flatulence-inducing sugars in chickpea milk.

## 7. Conclusions

In conclusion, the literature provides insights in terms of the various enzyme immobilisation systems and their use in the production of beverage products. It is evident that enzyme immobilisation systems have gained widespread popularity for beverage processing applications. They represent highly favoured solutions for eliminating the need for costly and time-consuming heat treatments to inactivate enzymes, which can also compromise the product quality. Beyond this important advantage, these systems offer uncountable benefits, not only for technological and processing improvements but also for enhancing the products’ sensory and nutritional attributes. They exhibit high pH and temperature stability across diverse conditions, enable multiple reuses of catalysts, simplify the removal of enzymes from the liquid medium, facilitate reaction control and reuse, and mitigate the risks of product contamination. Additionally, they enable the application of enzymes in different reactors and operations. The use of cross-linking agents was demonstrated to be advantageous by preventing enzyme leakage from the matrix material and ensuring maximal reuse capability. Furthermore, the utilisation of synthetic and inorganic materials in the development of immobilisation systems provides advantages in terms of optimised temperature and enhanced enzymatic stability, while safeguarding against adverse effects on the enzymes’ kinetic properties. Although the use of inorganic and synthetic materials increases the cost of the immobilisation process, in some cases they yield a higher maximum enzymatic rate and improved substrate–enzyme affinity. Additionally, the use of such materials contributes to reduced production times, preventing microbiological growth and guaranteeing food safety. Lastly, regardless of whether the carrier material is organic, inorganic, or synthetic or the immobilisation method employed in developing an enzyme immobilisation system, it is essential that the system is optimised to ensure peak performance, consistency, and maximum benefits offered by these innovative technologies. By tailoring the immobilisation process to meet the specific requirements of the application, it becomes possible to obtain the full potential of the enzyme immobilisation system, thereby enhancing the efficiency, product quality, and consumer satisfaction.

## 8. Future and Prospects

Extensive research studies have explored new supports and ways to immobilise enzymes, aiming to understand the behaviours of enzymes in both free and immobilised forms, focusing on their chemical and kinetic properties, as well as their operational stability. While these studies have been valuable, establishing general guidelines for enzyme immobilisation would greatly enhance this process and the utilisation of immobilised enzyme systems. Additionally, investigating how matrix materials interact with enzymes and how immobilisation affects the catalytic performance could be useful. Such information would guide the selection of appropriate immobilisation methodologies and parameters to obtain enzyme immobilisation systems suitable for the desired beverage. Developing food-grade enzyme immobilisation systems with non-toxic cross-linking agents, for instance, would also be beneficial for enhancing their use in food and beverage production applications.

While many studies have looked at small substrates such as lactose and phytic acid, there is a need to explore the potential of enzyme immobilisation systems dedicated to the treatment of large substrates such as starch and proteins. These polymeric substrates may show restricted diffusion into the enzyme immobilisation systems, which could result in difficulties in enzyme–substrate interactions, particularly for encapsulated systems. Studying changes in specificity due to immobilisation could also lead to exciting outcomes, such as the production of hydrolysates with varied nutritional and sensory properties, improving the food product quality and potentially producing different bioactive compounds. Lastly, integrating enzyme immobilisation systems and continuous processing technology holds tremendous promise for the future of the beverage production industry, as combining these approaches presents significant opportunities to enhance the process efficiency, consistency, and adaptability, ultimately improving the beverage product quality and purity. Therefore, advancements in enzyme immobilisation have the potential to transform the food industry, meeting consumer demands while reducing costs and minimising the environmental impact.

## Figures and Tables

**Table 1:** A comparison of pH behaviours between the immobilised and free forms of certain enzymes. The enzyme type and source, immobilisation method, carrier material, substrate, and beverage application are also listed.

Enzyme and Source	Method	Carrier/Support	Substrate	Optimum pH	pH Range/Stability	Application	Ref.
Tannase (*Penicillium rolfsii*)	Entrapment	Calcium alginate beads	Pectin	Increased from 4.0 to 4.3	Over 50% activity at all pH values tested for up to 16 h.	Apple juice clarification.	[21]
α-Acetolactate decarboxylase	Entrapment	Alginate gel beads	Z-Gly-Pro-pNA *	Increased from 4.5 to 5.5	86% activity preserved over pH range of 3.5 to 7.0.	Reduce off flavour and shorten beer maturation time.	[27]
β-Galactosidases (*Aspergillus oryzae*)	Entrapment	Barium alginate beads	ONPG *	Decreased from 4.5 to 4.0	Over 50% activity at pH range of 6.0 to 8.5.	Cow milk lactose hydrolysis.	[28]
Pectinase (*Aspergillus aculeatus*)	Covalent attachment	Glutaraldehyde-cross-linked alginate montmorillonite beads	Pectin	Decreased from 5.5 to 5.0	Higher pH stability than free enzyme in acidic conditions.	Pineapple juice clarification.	[29]
Glucose oxidase (*Aspergillus niger*)	Encapsulation	Calcium alginate beads	Glucose	Decreased from 5.5 to 4–4.5	Double activity remained compared to free enzyme at pH 3.0.	Reduce fermentable sugars in simulated wine musts.	[30]
Pectinase (*Aspergillus aculeatus*)	Entrapment	Calcium alginate beads	Pectin	Decreased from 5.0 to 3.0	95% and 79% activity at pH 4.0 and 7.0, respectively.	Apple and umbu juice clarification.	[19]
α-Galactosidase (*Debaryomyces hansenii*)	Adsorption	Cellulose film	pNPG *	Decreased from 5.0 to 4.0	60% activity over pH range of 4.0 to 6.5.	Soymilk RFO removal.	[31]
Protease (*Penaeus vannamei*)	Adsorption	Chitosan nanoparticles	Casein	Increased from 7.0 to 8.0	30% and 64% activity at pH 3.0 and 12.0, respectively. No activity for free enzyme.	Pomegranate juice clarification.	[32]
Exo-polygalacturonase (*Penicillium paxilli*)	Covalent attachment	Polyaldehyde dextran-cross-linked chitosan magnetic nanosupport	Pectin	Increased from 3.5 to 6.5	pH stability over a very broad range of pH values, mainly in acidic conditions.	Fruit juice clarification (apple, pineapple, pomegranate, grapes).	[33]
Lactase (*Escherichia coli*)	Covalent attachment	Glutaraldehyde-activated functional magnetic nanocomposite	ONPG *	Increased from 4.5 to 5.0	Higher activity levels than free lactase in both acidic and alkaline pH ranges.	Cow milk lactose hydrolysis.	[34]
Glyceraldehyde-3-phosphate dehydrogenase (*Lactobacillus plantarum*)	Covalent attachment	Amino-coated magnetic nanoparticles cross-linked with glutaraldehyde	Histamine	Increased from 6.5 to 7.5	80% enzymatic activity after 1 h of incubation over pH range of 4.5–8.5.	Histamine removal in winemaking process.	[35]
Xylanase (*Trichoderma longibrachiatum*)	Covalent attachment	Glutaraldehyde-activated silica gel support	Xylan	No change (pH 6.0)	Significant higher activity in acidic pH range.	Orange juice clarification.	[36]
Phytase (*Aspergillus niger*)	Adsorption	Zeolite modified with iron (II)	Phytate	No changes (pH 6.0)	Relative activity increased by 40% and 30% at pH 2 and 3, respectively.	Soymilk dephytination.	[37]
Pectinase (commercial preparation)	Covalent attachment	Polyaldehyde-pullulan-activated glass beads	Pectin or galacturonic acid	Increased from 5.0 to 5.5	Over 95% activity at pH range of 3.0 to 5.5.	Barberry juice clarification.	[38]
Pectinase (*Aspergillus tamari*)	Adsorption	Zr-treated pumice	Pectin	Increased from 6.0 to 7.0	Higher activity than free pectinase in the pH range of 7.0–9.0.	Fruit juice clarification.	[26]
Xylanase (*Bacillus pumilus*)	Covalent attachment	Glutaraldehyde-activated aluminum pellets	Xylan	Increased from 8.0 to 9.0	At pH 11.0, 48% and 72% activity (free and immobilised enzymes, respectively).	Grape and orange juice clarification.	[39]
Xylanase (*Mucor hiemalis*)	Covalent attachment	Genipin-activated alginate beads	Xylan	No changes (pH 5.0)	Over 80% activity over a pH range of 3.0–7.0.	Apple juice clarification.	[40]
β-Glucosidase (*Melaleuca pulchella*)	Adsorption	Monoaminoethyl–N-ethyl-agarose ionic support	pNPG *	No changes (pH 6.0)	70% activity at all acidic pH values and over 90% in neutral and basic pH ranges.	Grape juice and red wine clarification (anthocyanin hydrolysis).	[41]
α-Amylase (*Rhizoctonia solani*)	Covalent attachment	Chitosan beads cross-linked with glutaraldehyde	Soluble starch	Decreased from 5.5 to 4.5	70–80% activity after 5 days of tests at pH 5.5, 7.0, and 8.0.	Apple juice clarification.	[42]
Papain (*Carica papaya*)	Covalent attachment	Poly(HEMA) chitosan cryogels cross-linked with glutaraldehyde	Casein	No changes (pH 8.0)	A broader proteolytic activity profile than free enzyme.	Apple juice clarification via protein hydrolysis.	[43]
Pectinase and cellulase (commercial preparation)	Cross-linking	CLEA magnetic particles	Pectin	No changes (pH 4.0)	80% activity at pH 8.0.	Grape juice clarification.	[44]
Xylanase (*Bacillus pumilus*)	Covalent attachment	Glutaraldehyde aluminum oxide pellets	Xylan	No changes (pH 7.0)	More than two-fold activity at pH 4.0, 5.0, and 10.0.	Papaya juice clarification	[45]

* ONPG: o-nitrophenyl β-d-galactopyranoside; pNPG: 4-nitrophenyl β-D-glucopyranoside; Z-Gly-Pro-pNA: N-benzyloxy carbonyl-glycyl-prolyl-p-nitroanilide.

**Table 4:** Performance of enzymatic processes in continuous reactors regarding the enzyme type, immobilisation method, carrier or support material, reactor type, substrate, flow rate, hydrolysis conversion rate, and application.

Enzyme and Source	Method	Carrier/Support	Reactor Type	Substrate	Flow (mL/h)	Conversion	Application	Ref.
β-Galactosidase (*Kluyveromyces lactis*)	Covalent attachment	Glutaraldehyde–cotton cloth	Packed-bed reactor (pilot-scale)	Whole milk	840,000	60.5%	Whole milk lactose hydrolysis	[116]
					3,120,000	30.2%		
Pectinex Ultra SP-L (*Aspergillus aculeatus*)	Entrapment	Alginate beads	Packed-bed reactor	Apple juice	600	97.2%	Apple juice clarification	[19]
α-galactosidase (*Aspergillus oryzae*)	Entrapment	Glutaraldehyde–K-carrageenan	Fluidised-bed reactor	Soymilk	25	92.0%	RFOs soymilk hydrolysis	[55]
					50	85.0%		
α-Galactosidase (*Aspergillus oryzae*)	Entrapment	Glutaraldehyde–polyvinyl alcohol	Fluidised-bed reactor	Chickpea milk	30	94.0%	RFOs chickpea milk hydrolysis	[112]
					90	65.0%		
Naringinase (*Penicillium decumbens*)	Covalent attachment	Glutaraldehyde-modified zeolite	Packed-bed reactor	Grapefruit juices	0.25	54.0%	Debittering grapefruit juice	[107]
Lactase (*Pharmacopeia*)	Covalent attachment	Glutaraldehyde–glass beads	Packed-bed reactor	Whole milk	60	100.0%	Whole milk lactose hydrolysis	[108]
β-Galactosidase (*Aspergillus oryzae*)	Adsorption	Cross-linked concanavalin A-celite	Packed-bed reactor	Whole milk	20	95.0%	Whole milk lactose hydrolysis	[117]
					30	81.0%		
β-Galactosidase (*Kluyveromyces lactis*)	Covalent attachment	Modified collagen	Packed-bed reactor	Skimmed milk	120	75.0%	Skimmed milk lactose hydrolysis	[71]
β-Galactosidase (*Str. Thermophilus* and *L. bulgaricus*)	Entrapment	Barium alginate	Packed-bed reactor	Skimmed milk	6	92.9%	Skimmed milk lactose hydrolysis	[118]
					15	73.1%		
β-Galactosidase (*Kluyveromyces fragilis*)	Covalent attachment	Epicholorohydrin-activated cellulose beads	Fluidised-bed reactor	Whole milk	120	65.0%	Whole milk lactose hydrolysis	[109]
α-Galactosidase (*Aspergillus terreus*)	Entrapment	Cross-linked concanavalin A-calcium alginate	Fluidised-bed reactor	Soymilk	40	85.0%	RFOs soymilk hydrolysis	[119]
					80	72.0%		
Commercial enzyme cocktail for juice clarification	Covalent attachment	Glutaraldehyde-activated chitosan	Fluidised-bed reactor	Orange juice	30	87.0%	Orange juice clarification	[6]
					60	57.0%		
Commercial enzyme cocktail for juice clarification	Covalent attachment	Glutaraldehyde-activated chitosan	Packed-bed reactor	Orange juice	30	83.0%	Orange juice clarification	[6]
					90	80.0%		
Pectinase (*Aspergillus niger*)	Covalent attachment	Glutaraldehyde loofah sponge	Packed-bed reactor	Orange juice	30 180	89.0% 30.0%	Orange juice clarification	[120]
Papain *(Carica papaya)*	Covalent attachment	Commercial chitosan beads	Packed-bed reactor	White wine	360	59 to 96	White wine turbidity removal	[121]
Papain *(Carica papaya)*	Covalent attachment	Commercial chitosan beads	Packed-bed reactor	White wine	360	14 to 68	White wine protein haze reduction	[121]

## Data Availability

The original contributions presented in the study are included in the article, further inquiries can be directed to the corresponding author.
